# Impact of the intronic RFC1 expansion size in CANVAS phenotype: an oculomotor study

**DOI:** 10.1007/s00415-025-13150-9

**Published:** 2025-06-03

**Authors:** Mathieu Dupré, Ruben Hermann, Léo Vidoni, Isabelle Quadrio, Philippe Latour, Fabien Subtil, Caroline Froment Tilikete

**Affiliations:** 1 Neuro-Ophthalmology Unit, Hôpital Neurologique Pierre Wertheimer, Hospices Civils de Lyon, 59 Boulevard Pinel, 69500 Bron, France; 2 Lyon Neuroscience Research Center, INSERM 1028 and CNRS UMR5292, 69675 Bron, France; 3 Université Claude Bernard Lyon 1, 69100 Villeurbanne, France; 4 Molecular Neurogenetics Unit, Biochemistry and Molecular Biology DepartmentCentre de Biologie et de Pathologie Est, Hospices Civils de Lyon, 69500 Bron, France; 5 Biostatistics Department, Hospices Civils de Lyon, 69003 Lyon, France; 6 UMR 5558, CNRS, Université Claude Bernard Lyon 1, 69100 Villeurbanne, France

**Keywords:** Ataxia, Vestibular, Cerebellar, Videonystagmography, VHIT, Eye movements

## Abstract

The identification of the RFC1 homozygous intronic expansion in cerebellar ataxia neuropathy and vestibular areflexia syndrome (CANVAS) highlighted that genetically determined CANVAS patients exhibit a wide range of clinical presentations and natural course. Previous studies suggested a link between disease severity and the size of the intronic expansion. The aim of our study was to obtain quantitative data related to vestibular and cerebellar impairments using oculomotor recordings to provide further evidence of a link between RFC1 intronic expansion size and the phenotype. This study recruited 26 genetically determined CANVAS patients in whom the size of the pathological intronic expansion was measured on both alleles. In addition to clinical data, we also recorded the Overall Neuropathy Limitation Scale (ONLS) and conducted objective oculomotor testing. According to the median expansion length on one allele, the patients were divided in a longer intronic repeat subgroup and a shorter intronic repeat subgroup. Given the homozygous nature of this disease, this analysis was carried out for the smallest and for the longest allele. We found for the smallest allele that vestibular deficit and cerebellar impairment were significantly more frequent and mean ONLS, smooth pursuit, pendular visually enhanced vestibulo-ocular reflex, and head impulse vestibulo-ocular reflex gains were significantly more impaired in the subgroup of patients with the long intronic repeat. This work provides objective evidence for a functional impact of the pathological intronic expansion size in CANVAS and highlights the interest of oculomotor assessment in research and clinical practice both for diagnostic and potentially prognostic purposes.

**Supplementary Information:**

The online version contains supplementary material available at 10.1007/s00415-025-13150-9.

## Introduction

The specific combination of late-onset slowly progressive cerebellar ataxia, bilateral vestibular areflexia, and axonal sensory neuropathy (CANVAS) has been clearly recognized in 2011 [1]. From an electrophysiological and pathological perspective the non-length-dependent sensory deficit corresponds to a neuronopathy [2, 3]*.* The phenotypic description was further refined in several cohort studies, which identified the frequent and early occurrence of chronic cough or dysautonomia [4–6] as well as familial forms, leading to the publication of clinical and electrophysiological diagnostic criteria in 2016 [7]*.* Cortese et al. [8] identified a bi-allelic intronic pentanucleotide repeat AAGGGn in the *Replication Factor Complex subunit 1* (*RFC1*) gene in 100% of patients with familial CANVAS and 92% of patients with sporadic CANVAS, defining CANVAS as a genetic disorder with an autosomal recessive transmission*.* Since then, the proportions of heterozygous carriers has been estimated as ranging from 0.7 to 6.5% among the general population, and prevalence of bi-allelic carriers from 1/10,000 to 1/400 [8, 9]*.*

Description of large cohorts of adult-onset ataxic patients with the abnormal intronic expansion in the *RFC1* gene have shown a panel of different clinical presentations, natural course, and severity [10–12]. While the neuronopathy, sometimes subclinical, is found in 100% of cases, vestibular and cerebellar involvement may be absent, occurring in 93% and 79% of cases, respectively [10]. Associated features such as parkinsonian signs, possible motor neuropathy [11, 13], and cognitive decline [14] are rarely described*.* In addition, the natural course of the disease and the severity of disability can vary among patients. Data available from genetically determined cohorts argue in favor of a sequential disorder, beginning with the neuronopathy that worsens over the years, followed by the development of bilateral vestibular deficit (which may be included in peripheral neuropathy since it corresponds to VIII ganglionopathy) and later of cerebellar impairment [15]*.* However, there is considerable heterogeneity among patients, particularly in terms of clinical severity. A previous study reported that the median age of onset of neurological symptoms was 55 years, ranging from 30 to 80 years*.* Of note, some patients remain paucisymptomatic with an isolated sensitive neuropathy, sometimes discovered incidentally, while at the same age other patients require a wheelchair since they present the complete triad of the disease [10].

The explanation of such phenotype diversity and clinical severity, as already observed in Friedreich’s disease [16], may rely on the number of AAGGG repeated expansion in *RFC1*, which is reported to vary between 249 and 3885 [10, 12]. This hypothesis was recently comforted by a study in a large cohort of patients in which the expansion size in *RFC1* was correlated with phenotypic severity [12]. The authors found that a longer expansion size of both alleles was associated with earlier age of onset, earlier development of disabling symptoms (dysarthria, dysphagia, loss of independent walking), and presence of vestibular and/or cerebellar involvement. They also found that a larger expansion of the shortest allele was associated with a more pronounced cerebellar atrophy on MRI. However, there was a lack of quantitative data in this study that mainly reported declarative time onset/duration of symptoms to evaluate the effect of expansion size. The only objective data were the clinical demonstration of vestibular or cerebellar signs associated to neuronopathy, and the measurement of cerebellar atrophy.

The main objective of this study was to provide additional evidence regarding the association between the length of the intronic pentanucleotide repeat in *RFC1*, the phenotype, and different severity markers. The originality of the present study lies in the systematic oculomotor recording to obtain precise and quantitative data on vestibular and cerebellar functions.

## Materials and methods

### Patients

This single-center (Lyon University Hospital), observational, and cross-sectional cohort study included genetically determined CANVAS patients; all presented with peripheral sensory neuropathy proven by electrophysiological findings attended a neuro-otology consultation and underwent an oculomotor recording as well as a specific evaluation of the length of the repeated expansion. All the patients included had a positive bi-allelic AAGGG_n_ expansion in the *RFC1* gene, defined herein as sizes of the detected bands on Southern Blot greater than 2 kb [17] (corresponding to 400 repetition units) without flanking PCR bands, nor long-range PCR bands, but RP-PCR (AAGGG) positive with typical saw-tooth pattern, according to the Cortese et al. [8] protocol. Moreover, the study population was adult onset (age at least 18 years) and had to complete during the inclusion period a standardized clinical examination, performed by a referring physician that included assessment of vestibular and cerebellar involvement through electrophysiological oculomotor recording. Patients under protective measures or with incomplete clinical or missing oculomotor data were excluded.

The cohort presented herein corresponds to all CANVAS patients genetically identified between January 2020 and July 2021 in our department, fulfilling the criteria listed above.

The primary endpoint was the association of the clinical and functional features with the length of the intronic AAGGG expansions in each allele. The secondary endpoint was the association between oculomotor recording data and the length of the intronic AAGGG expansions in each allele.

### Ethics

This protocol was approved by the institutional review board of the Hospices Civils de Lyon, classified as category 3 non-interventional (no. 19-170). All included patients were informed of the study and provided their consent. This study was carried out according to the MR004 criteria of the *Commission Nationale de l'Informatique et des Libertés (CNIL*; no. 19–387). Compliance with standards of research involving humans as subjects. All procedures performed in this study involving human participants were in accordance with the ethical standards of the institutional and/or national research committee and with the 1964 Helsinki Declaration and its later amendments or comparable ethical standards.

### Clinical and electrophysiological data

Demographic, clinical, and recorded oculomotor data were prospectively collected during the clinical examination. Demographic and clinical data were sex, age at examination, age of onset of gait instability and duration of gait instability, presence of vestibular deficit, cerebellar impairment, and cough.

The Overall Neuropathy Limitations Scale (ONLS) was used as the functional score [18]. The ONLS is a score frequently used in clinical practice, composed of 2 parts, one assessing the functional capacities of the upper limbs (scored out of 5) and the other the functional capacities of the lower limbs (scored out of 7). A total score is calculated by adding the 2 sub-sections (scored out of 12), with a high score corresponding to significant functional disability.

All patients had undergone an electrophysiologic examination by electromyography prior to this study, including at least a sensitive and motor study of a sural nerve and a sensitive study of an ulnar nerve. Axonal sensory neuropathy was reported in all patients in the cohort, with no motor involvement. Quantitative data being available for 25/26 patients are shown in Supplementary Table 1.

The oculomotor assessment was performed as follows: visually and vestibularly driven eye movement recordings were first carried out using a device comprising a computer-controlled rotating chair (Synapsys SA, Marseille, France) on which patients were seated, head maintained upright in a head rest. Eye movements were recorded by an infra-red camera (VNG Ulmer, Synapsys SA) mounted in front of the left eye, recording at a 60 Hz frequency. A cover could be placed or removed over the right eye, to plunge the patient in darkness or to allow vision. A calibration was performed by asking the patient to follow a series of visual targets located 1.8 m away. Smooth pursuit was triggered by a target moving 20 degrees horizontally and sinusoidally at a 0.30 Hz frequency for 5 periods. Pendular visually enhanced vestibulo-ocular reflex (VVOR) and vestibulo-ocular reflex (VOR) were stimulated by a pendular chair rotation (frequency of 0.25 Hz, period of 30 s with a maximum chair speed of 60°/s). VVOR was recorded first with the right eye mask removed and then VOR in total darkness. For caloric test, patients were in the supine position, head tilted downwards by 30° in order to place the axis of the lateral canals perpendicular to the horizontal axis. The bithermal caloric test was performed using 60 mL of cold (30 °C) and hot (44 °C) water alternately in each ear for 30 s. Induced nystagmus was then recorded until it was exhausted (minimum 90 s).

Head Impulse Test (HIT) was recorded with a lightweight portable video device (vHIT; Hardware: ICS Impulse, GN Otometrics, Taastrup, Denmark; Software: Otosuite^®^ Vestibular software) allowing to record eye and head movements. Head movements were recorded with a 9-axis motion sensor and eye movements with a high-speed infra-red camera monitoring the right eye, both mounted on a lightweight eye frame. The patients were sitting at 1.5 m from the wall and horizontal head impulses were performed by experienced examiner standing behind the patient. A minimum of 10 valid horizontal head impulses were performed in each direction (left and right) with a target speed > 200°/s.

The software calculated online the change in eye position and for HIT the head position over time, allowing offline calculation of smooth pursuit gain [(maximum eye velocity)/(maximum target velocity)]; pendular VVOR gain and VOR gain [(maximum eye velocity)/(maximum chair velocity)]; reflectivity of bithermal caloric test (summing the absolute value of the maximum velocity of the slow phase of nystagmus measured for cold and warm water irrigation of both ears); and HIT VOR gain (ratio of the area under the eye velocity and head velocity curve from 60 ms before peak head acceleration to the last value of 0°/s as the head returns to rest) with mean HIT between left and right stimulations calculated.

### Definition of vestibular deficit and cerebellar impairment

Based on standards of the devices used and the Bàràny Society criteria [19], a bilateral vestibular deficit was defined as HIT gain on both semi-circular lateral canals < 0.7, and/or pendular VOR gain ≤ 0.15, and/or reflectivity in caloric test ≤ 5°/s.

A cerebellar impairment was defined by the presence of dysarthria, and/or hypotonia, and/or adiadochokinesis, and/or specifics oculomotor signs such as downbeat nystagmus (DBN), and/or hypermetric saccades.

Intentionally, dysmetria was not considered as a specific cerebellar sign as it could be confused with a proprioceptive deficit.

### Genetic studies

Genetic studies of all included patients were carried out in the molecular biology laboratory of Lyon University Hospital, using the protocol by Cortese et al. [8] for the CANVAS diagnosis. In order to quantify the size of the expansion as accurately as possible, a Southern Blot was performed as a complement with the detailed protocol below. The sizes of the detected bands were determined for each patient and the size of the expansion was estimated using the formula: size of the expanded band in kb − 5 kb, according to Cortese et al. [8].

Genomic DNA was extracted from whole blood using the NucleoSpin Blood L kit (Macherey-Nagel, Düren, Germany) according to the manufacturer’s instructions. A total of 10 mg of genomic DNA were digested for one night at 37° with EcoRI (225 U) before electrophoresis with the ladder Hind III (New England Biolabs, sizes range from 2027 to 23,130 kb) on a 0.8% agarose gel; migration lasted one night at 27 V. After gel preparation (depurination, denaturation, neutralization), DNA was transferred to a positively charged nylon membrane (Hybond XL, Dutscher) by capillary transfer of DNA over one night and was linked by NaOH 0.4 M during 25 min. Probes were prepared by PCR amplification of a genomic fragment, which randomly incorporates a P32-labeled nucleotide (dCTP32), then purified by exclusion chromatography, and finally DNA extracted from salmon sperm was added. After 20 min at 65° in a hybridization solution, membranes were hybridized with the radioactive labeled probe, during one night at 65°.

Then, membranes were washed at 65 °C for 15 min in each solution: the first was composed of standard sodium citrate (SSC) diluted to 2 × (from a 20 × stock solution) with 0.1% sodium dodecyl sulfate (SDS), the second was SSC 1 × with 0.1% SDS, and the third was 0.2 × SSC with 0.1% SDS.

Finally, hybridized fragments were visualized by phosphor imaging on a Scanner Fujifilm FLA-7000 (Fujifilm). The auto-radiographic film marked with P32 by the hybridized probe, after a one-night exposure, was scanned by the Fuji film FLA7000 Scanner. The positions of the single-stranded DNA bands were then compared with molecular weight controls to estimate fragment size, with a size-dependent uncertainty (estimated at 0.1 kb for a fragment of less than 7 kb, 0.2 kb between 7 and 11 kb, and 0.3 above).

### Statistics

All patients were ranked in ascending order according to the length of their intronic pentanucleotide expansion assessed by Southern blot. We performed 2 parallel analyses for each patient, one based on the allele with the shortest expansion (called short allele) and the other on the allele with the longest expansion (called long allele). For both analyses, the patients were divided into a subgroup with a long intronic repeat (equal or above the median) and a subgroup with a short intronic repeat (below the median).

Categorical variables are expressed as count and percentage, while continuous variables are expressed as median with the interquartile range. Caloric reflectivity cannot be used as quantitative data and was only used to define vestibular deficit (≤ 5°/s).

Comparison between the two subgroups (longest versus shortest expansion on one allele) were performed by the Wilcoxon Mann–Whitney test and by the Fisher's exact test. When continuous variables were statistically different between subgroups (longest versus shortest expansion), a linear regression model according to the age at examination was performed in each subgroup. When the slope of the linear regression was not statistically different between the subgroups, a model analyzed the mean difference between subgroups adjusted on age. All tests were two-sided, with an alpha level of 0.05. Analyses were performed on R version 4.4.0 (Vienna, Austria).

## Results

### Demographic, clinical, and electrophysiological data of the cohort

A total of 26 patients (17 male and 9 female) were included in this study, whose median age at examination was 66.5 years (interquartile range 61.3–71.0). The median duration of gait instability was 6.5 years (interquartile range 4.0–9.8). An axonal sensitive neuropathy was present in all patients, without motor impairment. A bilateral vestibular deficit was found in 20 patients (76.9%), chronic cough in 21 (80.8%), and cerebellar impairment in 17 patients (65.4%) with constant cerebellar oculomotor signs. It is of note that oculomotor cerebellar signs were the only cerebellar sign for 3 of these 17 patients. The median ONLS was 3/12 (interquartile range 1.3–4.0).

The median expansion length of the long allele was 6 kb (interquartile range 4.9–6.9) and that of the short allele was 4 kb (interquartile range 3.4–5.3). The minimal and maximal expansion length in this cohort were 3 kb and 23 kb, respectively.

### Analysis according to the allele with the shortest expansion

Since the median expansion length on the shortest allele was 4 kb, two subgroups were accordingly determined: ≥ 4 kb and < 4 kb. There was no difference regarding age (*p* = 0.92) at examination between the two subgroups. Although there was a trend toward a younger age at onset and a longer duration of ataxic symptoms in the ≥ 4 kb subgroup, it did not reach statistical significance (*p* = 0.18 and *p* = 0.34, respectively).

The phenotype was significantly more severe in the ≥ 4 kb subgroup compared to the < 4 kb subgroup for the following variables: presence of vestibular deficit and cerebellar impairment, median total and inferior limbs (IL) ONLS, mean smooth pursuit gain, mean pendular VVOR gain, and mean HIT gain (Table 1). Comparison of clinical variables including cerebellar specific (hypotonia, adiadochokinesis, dysarthria, hypermetric saccades, and down beat nystagmus) and non-cerebellar specific characteristics (ataxia, dysmetria) is available in Supplementary Table 2.

**Table 1:** Subgroups comparison for analysis on the short allele

Subgroup comparison based on the shortest allele			
	< 4 kb (*n* = 13)	≥ 4 kb (*n* = 13)	*p*
Expansion size longest allele (kb)	4.7 (4.4–6.0)	6.7 (6.0–8.2)	
Expansion size shortest allele (kb)	3.4 (3.2–3.6)	5.3 (4.9–5.8)	
Demographics			
Sex (male)	7/13 (53.8%)	10/13 (76.9%)	0.41
Age at examination (years)	66.0 (61.0–75.0)	67.0 (63.0–71.0)	0.92
Age of onset gait instability (years)	63.5 (56.5–67.3)	59.0 (54.0–63.0)	0.18
Duration of gait instability (years)	4.0 (0–9.0)	9.0 (6.0–13.0)	0.34
Clinical examination			
Vestibular deficit	7/13 (53.8%)	13/13 (100%)	**0.01**
Cerebellar impairment	5/13 (38.5%)	11/13 (84.6%)	**0.04**
Chronic cough	11/13 (84.6%)	9/13 (69.2%)	0.64
Total ONLS	2.0 (0–3.0)	4.0 (3.0–5.0)	**< 0.01**
IL ONLS	1.0 (0–2.0)	2.0 (2.0–3.0)	**< 0.01**
Oculomotor recording			
Smooth pursuit gain	0.73 (0.53–0.79)	0.50 (0.37–0.66)	**0.03**
Pendular VOR gain	0.30 (0.19–0.37)	0.13 (0.07–0.36)	0.26
Pendular VVOR gain	0.79 (0.73–0.98)	0.42 (0.25–0.66)	**0.01**
HIT gain	0.74 (0.65–0.98)	0.21 (0.13–0.51)	**< 0.01**

Data are described as frequency (percentage) for categorical variables or median (interquartile, IQR) for continuous variables

*ONLS* Overall Neuropathy Limitations Scale, *IL* inferior limbs, *VOR* vestibulo-ocular reflex, *VVOR* visually enhanced vestibulo-ocular reflex, *HIT* head impulse test, values shown in bold correspond to significant results

The slope of the linear regression was then calculated according to age at examination for continuous variables, which showed significant differences in the two subgroups: total and IL ONLS, smooth pursuit gain, VVOR gain, and HIT gain. When the slope was not statistically different between the two subgroups, a model analyzed the mean difference between the subgroups, adjusted on age.

**Fig. 1:**
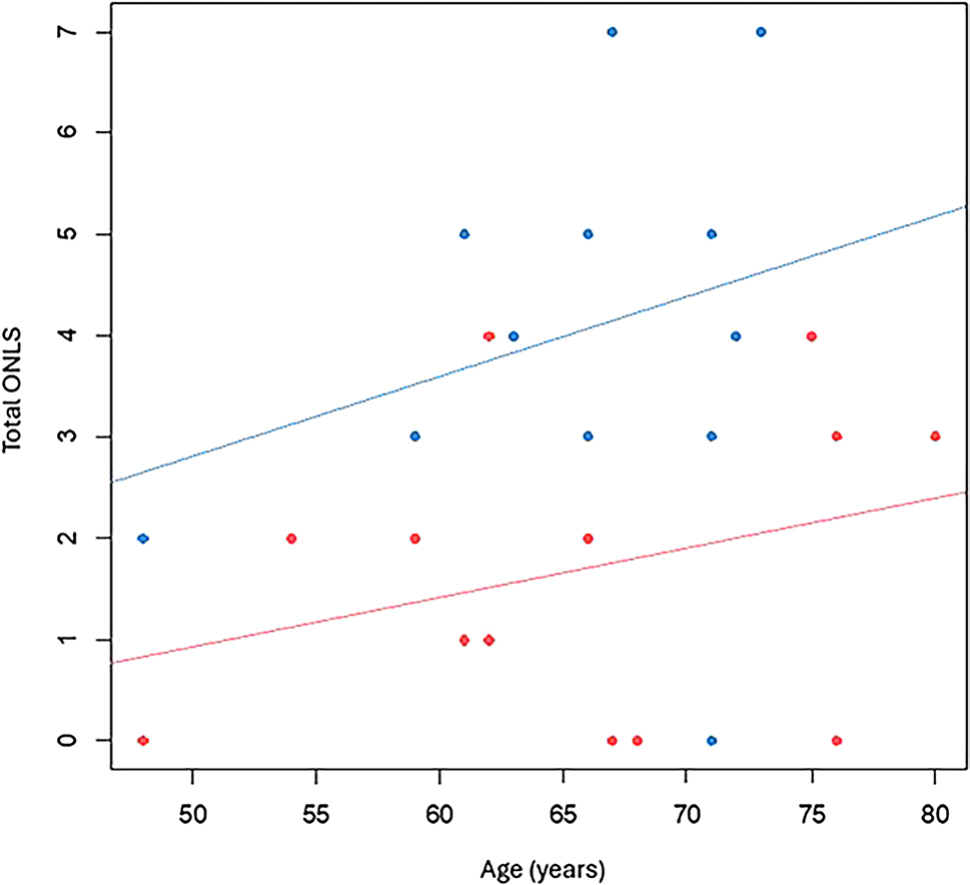
Total ONLS according to age at examination in the two patient subgroups based on the expansion size on the short allele. Red: patients with allele expansion size < 4 kb. Blue: patient with allele expansion size ≥ 4 kb. *ONLS* Overall Neuropathy Limitations Scale

The slope of the ONLS according to age was 0.049 in the < 4 kb subgroup (i.e., a gain of 0.049 ONLS points per year) and was not statistically different from the slope of 0.079 in the ≥ 4 kb subgroup (*p* = 0.737; Fig. 1). Considering equal slopes, we calculated a significant difference of 2.36 ONLS points at a given age between these 2 subgroups (95% CI [0.989, 3.734], *p* = 0.002).

There was no statistically significant difference regarding the change in slope between the < 4 kb subgroup and the ≥ 4 kb subgroup for the IL ONLS subcategory (slope value − 0.0049 in < 4 kb subgroup and 0.011 in ≥ 4 kb subgroup, *p* = 0.515), smooth pursuit gain (slope value < 0.01 in both subgroups, *p* = 0.227), and VVOR gain (slope value − 0.01 in < 4 kb subgroup and 0.01 in ≥ 4 kb subgroup, *p* = 0.094).

Considering equal slopes, a significant difference was found at a given age between these 2 subgroups of 1.52 ONLS points (95% CI [0.628, 2.424], *p* = 0.002), 0.158 smooth pursuit gain (95% CI [− 0.289, − 0.028], p = 0.019), and 0.305 VVOR gain (95% CI [− 0.533 − 0.028], *p* = 0.011).

**Fig. 2:**
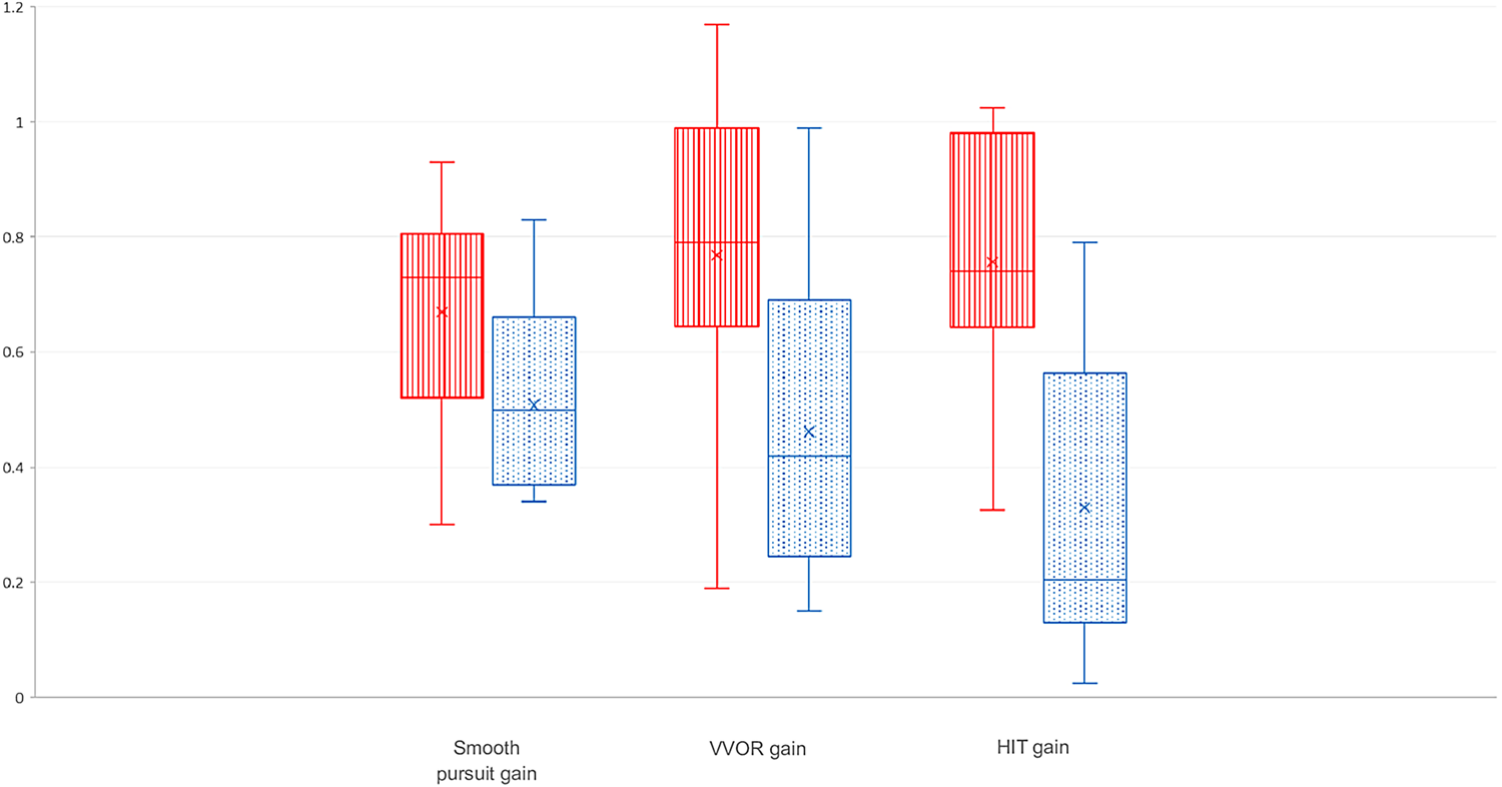
Box plot of oculomotor recording data in the two patient subgroups depending on the expansion size on the short allele. Red (vertical stripes): patients with allele expansion size < 4 kb. Blue (dotted): patient with allele expansion size ≥ 4 kb. *VVOR gain* visually enhanced vestibulo-ocular reflex gain, *HIT gain* head impulse test gain

For HIT gain, a difference at the limit of significance was found between the change in slopes (*p* = 0.059), which did not prompt the use of a model calculating an age-adjusted difference (Fig. 2).

### Analysis according to the allele with the longest expansion

Since the median expansion length of the longest allele was 6 kb, two subgroups were accordingly determined: ≥ 6 kb and < 6 kb. There was no significant difference of age at examination, age at onset, and of duration of ataxic symptoms between the two subgroups (*p* > 0.05).

The only significant difference between the two subgroups was the head impulse VOR gain, which was significantly lower in the ≥ 6 kb subgroup than in the < 6 kb subgroup (*p* = 0.02). Nevertheless, as in the results of the analysis based on the short allele, a difference at the limit of significance was found between the change in slopes of the two subgroups (*p* = 0.06), which did not prompt the use of a model calculating an age-adjusted difference. These data are shown in Supplementary Tables 3 and 4.

## Discussion

The aim of this prospective study was to provide objective evidence to the hypothesis regarding the implication of the length of the pentanucleotide RFC1 intronic expansion, on the phenotype and severity markers in CANVAS. To this end we used oculomotor recording to quantify cerebellar and vestibular impairment, typically present in CANVAS, in addition to usual clinical and functional assessment in an observational cross-sectional study.

Our main results were only observed in the analysis based on the allele with the shortest expansion length. While there was no difference in mean age of patients at time of examination in both subgroups, the subgroup of patients with expansion ≥ 4 kb showed increased disability, as measured with ONLS as well as significantly more frequent vestibular deficit and cerebellar impairment, decreased gains in smooth pursuit, VVOR, and HIT in comparison to the subgroup with expansion < 4 kb. These results suggest that the severity of the disease is linked to the increased size of at least the shortest allele expansion. Further analyses of the quantitative data (ONLS and different gains in eye movements) in both subgroups according to age at time of examination allowed us to find similar changes in slope over time; a significant and constant difference in severity was found in both subgroups. These results are concordant with those of Currò et al. [12] showing that the expansion size of both alleles, but predominantly the shortest, is associated with earlier age of onset and earlier development of disabling symptoms (dysarthria, dysphagia, loss of independent walking).

Studies of genetically determined CANVAS cohorts carried out to date highlighted both clinical heterogeneity and disease progression, which begin with a sensory neuronopathy that may extend to a vestibular nerve ganglionopathy, explaining the vestibular deficit. The progressive cerebellar syndrome appears to occur secondarily, often impacting functional status, such as walking ability or dysarthria [10–12]. The originality of our work was the reliable and reproducible investigation of vestibular and cerebellar function using eye movements recording. Indeed, while neuro-otologists first described CANVAS as the association of deficit of vestibular and cerebellar control of slow eye movements [20, 21], much recent cohorts does not precisely report these impairments. In our cohort, we studied smooth pursuit that physiologically reflects visually guided slow eye movements in which the cerebellum plays a predominant role; pendular and head impulse induced vestibulo-ocular reflex, assessing the vestibular system at medium and high frequencies, respectively; and visuo-vestibulo-ocular reflex (VVOR), assessing the control of both vestibular and cerebellar functions on slow eye movements. Of note, VVOR deficit is relatively specific to CANVAS [22]. This reflex is assessed during a rotatory chair test, with vision allowed. In case of isolated vestibular impairment, visually guided slow eye movements depending on cerebellar functions (underlying smooth pursuit in particular) compensate for this deficit. Conversely, in case of an isolated oculomotor cerebellar impairment, vestibular functions help maintain gaze stabilization. For over 20 years, VVOR involvement has been described as relatively specific to CANVAS, since it presupposes combined cerebellar and bilateral vestibular impairment. Remarkably, all these parameters appear in our work to be significantly associated with the expansion length on the shortest allele, therefore showing that quantitative oculomotor assessment is a reliable marker of functional impairment in CANVAS and prognostic. It is worth noting the great difference between HIT gain in the ≥ 4 kb expansion subgroup and the < 4 kb expansion subgroup. HIT evaluates RVO at high frequency that corresponds to its real-life function in stabilizing gaze and posture during locomotion. Therefore, a loss of 0.55 HIT gain is very clinically relevant in terms of functional prognosis of the disease.

In our study, we added ONLS as a functional score*.* Since progressive postural ataxia and walking difficulties in CANVAS result from sensory neuronopathy that can be associated over time with vestibular deficit and cerebellar involvement, evaluating a functional score in such a multisensory and organ-related postural dysfunction is complex. The ONLS is validated in peripheral neuropathies [18], which represent the core and constant impairment of CANVAS. This score was therefore preferred to specific cerebellar syndrome scales since it is easier to use and broadly assess of functional impairment. Interestingly, we found a significant effect of the length of the shortest allele on the total score and the inferior limb score.

Disease onset of CANVAS can also result in other various symptoms including pain, dysesthesias, and chronic cough [10, 11]. Deciding which symptoms might be used to date the exact time of disease onset is also crucial. In Currò et al*.* [12], the authors chose to collect any neurological symptoms including sensory symptoms, dysarthria, dysphagia and oscillopsia, and use of walking aids but did not to take chronic cough into account, which can occur several years before the first sensitive or ataxic symptoms in CANVAS patients. Herein, we found challenging for the patients to be precise about subjective sensory symptoms; we therefore decided to define the disease onset as the point when the patient first reported ataxic symptomatology. This methodological difference, as well as the size of our cohort, may explain why no significant effect of the allele expansion on the mean age of onset of gait instability and mean duration of gait instability, as reported by Currò et al.

Previous genetic studies have mentioned microsatellites, also called short tandem repeats (STRs), corresponding to small DNA repeats (classically between 5 and 50 repeats) of typically 2 to 5 bp in length scattered in the human genome, and which could have an important function in the regulation of gene expression [23]. In addition, there is a threshold below which high variability in STR repeat number has a low pathophysiological impact [24]. Exceeding this threshold of repeat expansions results in several dozen of genetic diseases, the majority of which involve the nervous system, and particularly the cerebellum [25, 26]. This threshold has not yet been formally established for CANVAS, but is estimated around 250 repeat units according to the Currò et al*.* study [12].

The pathophysiological mechanisms related to repeated expansions essentially include RNA toxicity (notably by *foci* deposition), protein toxicity, and loss or gain of function of the encoded protein [24]. Although the recessive mode of transmission suggests a loss of function, as in Friedreich's disease (also due to intronic repeated expansions) [27], this has not been directly demonstrated in CANVAS yet. No qualitative (via RNA sequencing) or quantitative (via quantitative reverse transcription-PCR) alterations were found in *RFC1* transcript or its satellite genes. The pathological studies found no endogenous RNA *foci*. Furthermore, a comparative study of RFC1 final protein levels by Western blot, both in peripheral tissues and in the brain, revealed no significant difference [8]. Nevertheless, in the case of CANVAS, our work confirms the functional impact of the repeated expansion size, which, along with the recent identification of truncating variants in compound heterozygotes [28–30], is strong argument for the loss-of-function hypothesis.

The limitations of our study rely firstly in its single-center and cross-sectional design that limits the extrapolation of our findings due to the small sample size. In addition, a selection bias due to the recent identification of the genetic abnormality in CANVAS may have led to include most severe patients; those with the most suggestive clinical signs were firstly genetically assessed. Also, our genetic method of diagnostic does not allow to take into account rare and recently reported population-specific configurations, or heterozygous compound with truncating variants [28–32]. Despite the small sample size, the statistical method allowed to split the 26 patients in two equal-size subgroups according to the median of the expansion size, for two parallel analyses: one for the allele with the shortest expansion length and one on the allele with the longest expansion length. This allowed us to compare subgroups of patients according to the size of the short allele expansion or of the long allele expansion. Moreover, given the panel of age of patients at time of examination, we finally used a linear regression model according to age at examination time and compared them between subgroups of patients.

This work provides objective evidence for a functional impact of the pathological intronic expansion length in CANVAS and for the pathophysiological hypothesis of a loss-of-function mechanism. Furthermore, our study highlights the interest of oculomotor assessment both for diagnostic and potentially prognostic purposes.

## Supplementary Information

Below is the link to the electronic supplementary material.

Supplementary file1 (DOCX 15 KB)

Supplementary file2 (DOCX 16 KB)

Supplementary file3 (DOCX 16 KB)

Supplementary file4 (DOCX 16 KB)

## Data Availability

The data are available from the corresponding author upon reasonable request.
