# Supplementary material for: Impact of the intronic RFC1 expansion size in CANVAS phenotype: an oculomotor study
Source: J Neurol. 2025 Jun 3;272(6):442. doi: 10.1007/s00415-025-13150-9 (PMC12134041; doi:10.1007/s00415-025-13150-9)
Supplement: Supplementary file 1 — Supplementary file1 (DOCX 15 KB) [file 415_2025_13150_MOESM1_ESM.docx]

|  | Sensory nerve conduction^a^ | | Motor nerve conduction^a^ | Myography | Conclusion |
| --- | --- | --- | --- | --- | --- |
| Subject | Sensory ulnar amplitude (µV)  N>18 | Sensory sural amplitude (µV)  N>10 | Motor sural amplitude (mV)  N>4 |  |  |
| 1 | 0,0 | 0,0 | 12,5 | Normal | Axonal sensitive neuropathy |
| 2 | 1,6 | 0,0 | 5,8 | Normal | Axonal sensitive neuropathy |
| 3 | 1,0 | 0,0 | 21,9 | Normal | Axonal sensitive neuropathy |
| 4 | NA | NA | NA | NA | Axonal sensitive neuropathy |
| 5 | 4,0 | 1,0 | 8,2 | Normal | Axonal sensitive neuropathy |
| 6 | 4,4 | 1,0 | 14,8 | Normal | Axonal sensitive neuropathy |
| 7 | 0,0 | 0,0 | 8,8 | Normal | Axonal sensitive neuropathy |
| 8 | 0,0 | 0,0 | 14,4 | Normal | Axonal sensitive neuropathy |
| 9 | 0,0 | 0,0 | 10,6 | Normal | Axonal sensitive neuropathy |
| 10 | 0,0 | 0,0 | 11,3 | Normal | Axonal sensitive neuropathy |
| 11 | 0,0 | 0,0 | 15,7 | Normal | Axonal sensitive neuropathy |
| 12 | 0,9 | 0,0 | 8,6 | Normal | Axonal sensitive neuropathy |
| 13 | 8,0 | 0,4 | 9,3 | Normal | Axonal sensitive neuropathy |
| 14 | 24,0 | 5,0 | 19,9 | Normal | Axonal sensitive neuropathy |
| 15 | 0,0 | 0,0 | 12,8 | Normal | Axonal sensitive neuropathy |
| 16 | 0,0 | 0,0 | 10,2 | Normal | Axonal sensitive neuropathy |
| 17 | 6,0 | 4,0 | 10,7 | Normal | Axonal sensitive neuropathy |
| 18 | 0,0 | 0,0 | 16,0 | Normal | Axonal sensitive neuropathy |
| 19 | 0,0 | 0,0 | 6,5 | Normal | Axonal sensitive neuropathy |
| 20 | 2,0 | 2,0 | 17,0 | Normal | Axonal sensitive neuropathy |
| 21 | 2,0 | 0,0 | 7,2 | Normal | Axonal sensitive neuropathy |
| 22 | 9,0 | 1,0 | 8,1 | Normal | Axonal sensitive neuropathy |
| 23 | 0,0 | 0,0 | 6,6 | Normal | Axonal sensitive neuropathy |
| 24 | 0,0 | 0,0 | 5,6 | Normal | Axonal sensitive neuropathy |
| 25 | 10,0 | 0,0 | 7,5 | Normal | Axonal sensitive neuropathy |
| 26 | 0,0 | 0,0 | 13,7 | Normal | Axonal sensitive neuropathy |

**Supplementary Table 1. Peripheral electroneuromyography data**

*Abbreviations : N: normal ; NA: not available*

*^a^Antidromic technique was used for sensory and motor nerve conductions*
