# Supplementary material for: Impact of the intronic RFC1 expansion size in CANVAS phenotype: an oculomotor study
Source: J Neurol. 2025 Jun 3;272(6):442. doi: 10.1007/s00415-025-13150-9 (PMC12134041; doi:10.1007/s00415-025-13150-9)
Supplement: Supplementary file 2 — Supplementary file2 (DOCX 16 KB) [file 415_2025_13150_MOESM2_ESM.docx]

| ***Subgroups clinical comparison based on the allele with the shortest expansion*** | | | |
| --- | --- | --- | --- |
|  | <4kB (n=13) | ≥4kB (n=13) | p |
| **Clinical examination** |  |  |  |
| Ataxia | 9/13 (69%) | 13/13 (100%) | 0.1 |
| Dysmetria | 6/13 (46%) | 11/13 (85%) | 0.1 |
| Hypotonia^a^ | 0/13 (0%) | 7/13 (54%) | **<0.01** |
| Adiadochokinesis^a^ | 2/13 (15%) | 4/13 (31%) | 0.64 |
| Dysarthria^a^ | 1/13 (8%) | 8/13 (61%) | **0.01** |
| **Oculomotor findings** |  |  |  |
| Down beat nystagmus^a^ | 2/13 (15%) | 4/13 (31%) | 0.64 |
| Hypermetric saccades^a^ | 2/13 (15%) | 5/13 (38%) | 0.38 |
|  |  |  |  |
| Cerebellar impairement | 5/13 (38%) | 11/13 (85%) | **0.04** |

**Supplementary Table 2. Subgroups comparison for clinical examination** **on the allele with the shortest expansion**

Data are described as frequency (percentage).

^a^Clinical features which have been considered specific for cerebellar involvement
