# Supplementary material for: Impact of the intronic RFC1 expansion size in CANVAS phenotype: an oculomotor study
Source: J Neurol. 2025 Jun 3;272(6):442. doi: 10.1007/s00415-025-13150-9 (PMC12134041; doi:10.1007/s00415-025-13150-9)
Supplement: Supplementary file 3 — Supplementary file3 (DOCX 16 KB) [file 415_2025_13150_MOESM3_ESM.docx]

| **Subgroups comparison for principal analysis on the allele with the longest expansion** | | | |
| --- | --- | --- | --- |
|  | <6kb (n=12) | ≥6kb (n=14) | p |
| Expansion size longest allele (kb) | 4.7 (4.3-5.5) | 6.8 (6.5-8.5) |  |
| Expansion size shortest allele (kb) | 3.6 (3.2-4.3) | 4.6 (3.8-5.3) |  |
| **Demographics** |  |  |  |
| Sex (male) | 9/12 (75.0%) | 8/14 (57.1%) | 0.43 |
| Age at examination (years) | 67.5 (65.0-73.5) | 64.5 (59.5-71.0) | 0.20 |
| Age of onset gait instability (years) | 63.5 (60.0-66.0) | 57.0 (51.0-63.0) | 0.16 |
| Duration of gait instability (years) | 6.5 (0-9.3) | 7.0 (4.0-10.5) | 0.45 |
| **Clinical examination** |  |  |  |
| Vestibular deficit | 7/12 (58.3%) | 13/14 (92.9%) | 0.06 |
| Cerebellar impairment | 5/12 (41.7%) | 11/14 (78.6%) | 0.10 |
| Chronic cough | 8/12 (66.7%) | 12/14 (85.7%) | 0.36 |
| Total ONLS^a^ | 1.5 (0-3.3) | 3.5 (3.0-4.8) | 0.08 |
| IL^b^ ONLS | 0.5 (0-2.3) | 2.0 (2.0-2.0) | 0.10 |
| **Oculomotor recording** |  |  |  |
| Smooth pursuit gain | 0.67 (0.53-0.78) | 0.48 (0.37-0.65) | 0.08 |
| VOR^c^ gain | 0.30 (0.18-0.38) | 0.17 (0.08-0.33) | 0.37 |
| VVOR^d^ gain | 0.76 (0.48-0.88) | 0.48 (0.30-0.75) | 0.57 |
| HIT^e^ gain | 0.74 (0.57-0.84) | 0.42 (0.14-0.62) | **0.02** |

**Supplementary table 3. Subgroups comparison for principal analysis on the allele with the longest expansion**

Data are described as frequency (percentage) for categorical variables or median (interquartile, IQR) for continuous variables.

^a^ONLS: Overall Neuropathy Limitations Scale;

^b^IL: inferior limbs;

^c^VOR: vestibulo-ocular reflex;

^d^VVOR: Visually enhanced Vestibulo-ocular Reflex

^e^HIT: Head Impulse Test
