# Supplementary material for: Impact of the intronic RFC1 expansion size in CANVAS phenotype: an oculomotor study
Source: J Neurol. 2025 Jun 3;272(6):442. doi: 10.1007/s00415-025-13150-9 (PMC12134041; doi:10.1007/s00415-025-13150-9)
Supplement: Supplementary file 4 — Supplementary file4 (DOCX 16 KB) [file 415_2025_13150_MOESM4_ESM.docx]

| ***Subgroups clinical comparison based on the allele with the longest expansion*** | | | |
| --- | --- | --- | --- |
|  | <6kB (n=12) | ≥6kB (n=14) | p |
| **Clinical examination** | |  |  |
| Ataxia | 9/12 (75%) | 13/14 (93%) | 0.31 |
| Dysmetria | 6/12 (50%) | 11/14 (79%) | 0.22 |
| Hypotonia^a^ | 2/12 (17%) | 5/14 (36%) | 0.39 |
| Adiadochokinesis^a^ | 4/12 (33%) | 2/14 (14%) | 0.36 |
| Dysarthria^a^ | 2/12 (17%) | 7/14 (50%) | 0.11 |
| **Oculomotor findings** |  |  |  |
| Down beat nystagmus^a^ | 2/12 (17%) | 4/14 (29%) | 0.65 |
| Hypermetric saccades^a^ | 1/12 (8%) | 6/14 (43%) | 0.08 |
|  |  |  |  |
| Cerebellar impairement | 5/12 (42%) | 11/14 (79%) | 0.1 |

**Supplementary table 4. Subgroups comparison for clinical exam on the allele with the longest expansion**

Data are described as frequency (percentage)

^a^Clinical features which have been considered specific for cerebellar involvement
